# Fetuin-A Modulates Tumor Growth and Invasion in a Basal-like Triple Negative Breast Cancer Cell line, MDA-MB-468

**DOI:** 10.26502/fjppr.0103

**Published:** 2025-02-04

**Authors:** Divya B Kenchappa, Olga Korolkova, Nobelle Sakwe, Peace Odiase, Michael G. Izban, Amos Sakwe, Josiah Ochieng

**Affiliations:** 1 Department of Biochemistry, Cancer Biology, Neuroscience and Pharmacology, Meharry Medical College, 1005 Dr. D.B. Todd Blvd., Nashville, TN 37208; 2 Present Address: Department of Oncology, Albert Einstein College of Medicine, Bronx, NY 10461; 3 Department of Pathology, Meharry Medical College, 1005 D.B. Todd Blvd., Nashville, TN 37208; 4 Department of Biomedical Science, Graduate School, Meharry Medical College, 1005 D.B. Todd Blvd., Nashville, TN 37208

**Keywords:** Fetuin-A, Triple Negative, Breast Cancer, TLR4, Invasion, Basal-like

## Abstract

The present studies were undertaken to address the innovative role of fetuin-A in the growth and invasion potential in a triple negative breast cancer (TNBC) cell line, MDA-MB-468. Basal like TNBC that express high levels of ectopic fetuin-A have poorer prognosis for the patients compared to those that express low levels of the protein. We overexpressed fetuin-A in MDA-MB-468 and then determined the invasive potential of fetuin-A overexpressing cells vs controls transfected with empty vector. We also determined the adhesion and growth potential of the cells in the presence of only fetuin-A in serum free medium and also in complete medium. Our data suggest that fetuin-A overexpression significantly enhances the invasive potential of the cells and also the expression of Toll like receptor 4 (TLR4) on these cells. More importantly, the cells rely on fetuin-A-TLR4 signaling network for growth and invasion because the specific TLR4 inhibitor CLI-095 (resatorvid) abrogates fetuin-A mediated growth and invasion. Taken together, the data suggest that fetuin-A-TLR4 signaling network plays a significant role in the growth and invasion potential of TNBC.

## Introduction

We previously demonstrated the significance of fetuin-A in the initiation and progression of mammary tumors using a transgenic mouse model for breast cancer. In this model system, we were able to demonstrate that lack of fetuin-A in the PyMT transgenic background significantly prolonged the latency period of mammary tumor development. Furthermore, tumors that formed in mice that lacked fetuin-A were much smaller in size with low number of proliferating cells [1]. However, this model was based on liver produced fetuin-A. In the present studies we considered not only the role played by fetuin-A in the medium supplied to the tumor, but also fetuin-A produced by the tumor (ectopic fetuin-A).

Fetuin-A is presently considered a multifunctional protein with a potential to play several roles in tumorigenesis [2]. It is a serum glycoprotein primarily synthesized and secreted by liver parenchymal cells. It has a molecular weight of approximately 50 kDa and is heavily glycosylated [3]. Whereas its main physiological function is inhibition of ectopic calcification, reports show that ectopic fetuin-A plays pivotal roles in the progression of lung cancer [4], glioblastomas [5], and prostate cancer [6]. It has also been shown as a potential biomarker for breast cancer [7] as well as hypo-pharyngeal squamous cell carcinoma [8]. We have reported that fetuin-A added to the tumor cells plays a scaffolding role on the surfaces of a prostate cancer cell line, LNCaP, where it is concentrated on the surfaces of the cells growing as spheroids and simultaneously trap extracellular vesicles such as exosomes to effectively mediate growth [9]. Our studies suggested that this could be a contributing pathway for growth in prostate cancer cells particularly those that do not rely entirely on androgen signaling for their growth [9]. We questioned whether the same growth strategy could be employed by breast cancer cells especially those that lack receptors for estrogens, progesterone and epidermal growth factor 2, commonly known as triple negative breast cancer (TNBC). Whereas the other subtypes of breast cancer such as luminal A and B rely on estrogens or progesterone for growth, TNBC could rely on other pathways such as those propagated by fetuin-A and epidermal growth factor 1 to propel their growth [10]. On tumor cells, we have identified TLR4 as the receptor with which fetuin-A interacts to promote tumor growth [11]. Of note is the plethora of studies that have implicated TLR4 in the progression of tumor cells [12–14]. For many of these studies, it was assumed that the ligand for TLR4 activation even on tumor cells was the endotoxin, lipopolysaccharide (LPS). This signaling network was then responsible for chronic inflammatory conditions that promoted tumor progression [13, 15, 16]. Indeed, LPS as ligand for TLR4 activation during tumor progression has been validated in gastric tumors [17]. However, in other tumors linking LPS in the activation of TLR4 on tumor cells was not possible suggesting there are other endogenous ligands that activate TLR4 on tumor cells [13].

Here we have questioned the potential role of fetuin-A to modulate through TLR4, the growth and invasive capacity of a TNBC cell line, MDA-MB-468. The cell line was derived from a pleural effusion metastasis of a breast cancer patient [18]. It exhibits phenotypes that resemble basal-like breast cancer, a sub-type typically associated with TNBC. The cells are highly proliferative and invasive [19] and have been instrumental in advancing our knowledge of TNBC as well as the development of novel treatment strategies for this challenging disease [20]. The impetus for the present studies were analysis of breast cancer survival data Cancer Genome Atlas, (TCGA) [21]. In these studies, basal-like as well as basal-like triple negative breast cancer cells that expressed high levels of fetuin-A had significantly poor overall survival compared to those that expressed low levels of the protein. This was not replicated in both luminal A and luminal B sub-types which are the most common breast cancer sub-types [21]. The studies underscored the significance of ectopic fetuin-A in the progression of breast cancer.

## Materials and Methods

### Cells

MDA-MB-468 was purchased from ATCC, Manassas, Va. The cells were cultured in L15 (Leibovitz’s) medium supplemented with 10% fetal bovine serum, 1% NaHCO3 and 1x antibiotic/antimycotic (Life Technologies, Grand Island, NY, U.S.A). The cells were maintained in humidified CO2 incubator at 37°C. The serum-free medium (SFM) used in the studies contained 1% (w/v) bovine serum albumin.

### Over expression of Fetuin-A (FA) by Lentiviral method

In order to confer stable overexpression, a lentiviral vector was used for gene delivery. In brief, HEK 293FT cells with a low passage number were seeded in 10-cm plates at a density of 4.0 × 106 cells and allowed to achieve 80% confluency overnight. On the next day, 2 h before transduction, the medium was replaced with 10 ml fresh, pre-warmed DMEM containing 10% FBS. A total of 20 µg of DNA plasmids (10 µg of flag-tagged Fetuin-A-expression vector and 10 µg of psPAX2 packaging and pMD2. G envelope plasmid DNA) were combined in 1 ml serum-free, antibiotic-free DMEM in a 10-cm plate. The control plates comprised GFP-containing vectors. Then, the cells were transduced using OriGene TurboFectin 8.0 Transfection Reagent (Cat # TF81005). The medium was removed around 14–16 h after transduction, and 8 ml/dish fresh preheated DMEM containing 10% FBS was added. After 12 h, the supernatant was collected, and this procedure was continued for 72 h at 8–12 h intervals. When all the supernatants were collected, centrifugation was performed at 1,500 rpm for 5 min; the supernatant was filtered through a 0.45-µm filter. The viral titer was detected using qPCR Lentiviral titer kit (LV900) from abm. The filtered supernatant was aliquoted and stored at −80°C. MDA-MB-468 cells were plated in 6 well plates at a density of 0.5 × 105 cells per well 24 h prior to viral infection and grown at 37°C with 5% CO2 overnight. On the day of infection, lentiviral particles were added to cells with the mixture of complete media and 8 µg/mL polybrene. The cells were incubated with virus for 8 hours, and then medium containing FBS was added. Second round of infection was done for next 8 hours and media was changed. Forty-eight hours later, the infected cells were subjected to selection with 2 µg/mL puromycin for 1 week.

### Reverse Transcriptase and Real-Time PCR

MDA-MB-468 cells were grown in 10 cm dishes and cultured in complete medium containing 10% FBS. Total RNA was isolated from the cells using RNeasy MiniKit from QIAGEN (Valencia, CA). For the single-strand cDNA synthesis, 1 μg of total RNA was reverse-transcribed using High-Capacity cDNA Reverse Transcription Kit according to manufacturer’s protocol from Thermofisher Scientific (Catalog number: 4368814). Real time PCR was performed using Fetuin-A Taqman gene specific assays (Catalog number: Hs00155659_m1) and TaqMan^™^ Fast Advanced Master Mix (Catalog number: 4444556). Beta Actin was used as endogenous controls. The expression levels of the genes were normalized against those of the endogenous control using the 2−ΔΔCt method, and p <0.05 considered statistically significant (*p <0.05; **p <0.01;***p <0.001).

### Western Blot Assays

Western blotting was done following standard protocols. Proteins were separated on SDS-polyacrylamide gels and transferred onto nitrocellulose membranes. Membranes were blocked with 5% non-fat milk and incubated with the primary antibodies AHSG (R&D, Cat # MAB 1184), anti-flag (Sigma, Cat # F3165), Actin (Cell Signaling, Cat # 4967) overnight at 4°C. Membranes were washed and incubated with horseradish peroxidase-conjugated secondary antibodies at room temperature. The antigen-antibody complexes were detected using enhanced chemiluminescence detection reagents. Protein bands were imaged using a ChemiDoc gel imaging system.

### Immuno-histochemistry Assays (IHC)

Control (MDA-MB-468EV) and fetuin-A over-expressing cells were seeded at an appropriate density and allowed to attach to the slide (30,000 cells/chamber in an 8-chambered glass slide). Cells were plated one day before staining to achieve 60–80% confluency. The following day cells were fixed in 10% Formalin for 5 min at room temperature and gently washed in PBS 3 times (5 min/wash). Immunostaining was performed on Labvision Autostainer using the Ultra-vision Quanto (HRP Polymer DAB) Detection System (Thermo Scientific) using the default detection system protocol with a 45 min antibody incubation step. Primary mouse monoclonal anti-human fetuin-A/AHSG antibody (R&D Systems, MAB1184) was used at a dilution of 1:500 in Antibody OP Quanto antibody diluent (Thermo Fisher, TA-125-ADQ). The Quanto DAB Plus system was used (5 min) for color development. The slides were counterstained with Mayer’s hematoxylin (1 min), dehydrated, and cover-slipped using cytoseal XYL (Thermo Fisher). Staining control slide lacked primary antibody. Images were captured using an Olympus BX41 microscope equipped with a Motic 5 MP digital webcam.

### Invasion Assays

The assay was done essentially as previously described [11]. The same number of cells (125,000 cells/chamber) in SFM were added to the upper chambers (500 µl) of Boyden Chamber trans-well. The bottom chambers contained 500 µl of L15 medium containing 10% fetal bovine serum (FBS)-complete medium (CM). As controls, some of the bottom wells contained serum free medium containing 1% (w/v) BSA. After 24 h, the invading cells migrated to the underside of the polycarbonate filters with Matrigel coating. After removing the cells remaining in the upper chambers with cotton swabs, the invading cells were fixed in 4% formalin, washed in PBS and then stained with crystal violet as described [11], and viewed under a microscope. The number of cells in 20X field of view were counted and expressed as mean + S.D. (N = 6).

### Regulation of TLR4 surface expression by Fetuin-A

MDA-MB-468 cells are reported to express very low levels of TLR4, almost negligible [22]. We questioned whether fetuin-A could modulate cell surface expression of TLR4 in these cells, either upregulation or downregulation. The cells (MDA-MB-468, MDA-MB-468-EV and MDA-MB-468FA) were incubated at 37°C in humidified CO2 incubator until approximately 80% confluent. They were then washed in warm PBS and detached with 2 mM EDTA in PBS. The cells were pelleted and re-suspended as single cells in cold FACS buffer (PBS containing 1% BSA) in Eppendorf tubes (1 × 106 cells per tube). To determine the influence of added fetuin-A on cell surface TLR4 expression, the cells were suspended in FACS buffer containing fetuin-A (2 mg/ml). The tubes were incubated without (unlabeled controls) or with Cy3-labeled anti-human CD284 (TLR4 antibody; Bio-Legend, San Diego, CA USA; 10 µl per tube) for 30 min at 4°C with end on end rotation. The cells were then washed 3x with cold FACS buffer and analyzed on a Cell-Stream flow cytometer (Luminex) using channel D4 with excitation wavelength 532 nm, and emission 583/24 nm.

### 2-Dimensional (2-D) and 3-Dimensional (3-D) growth of MDA-MB-468 cells in the presence of medium containing none (SFM) or either fetuin-A (2 mg/ml) or fetal bovine serum (CM) (10% v/v)

The MDA-MB-468 parental cell lines were plated quadruplicates either in treated 96-well culture plates (2D) or ultra-low attachment plates (3-D) at 2 × 104 cells/well in 200 µl of medium. Cell growth was evaluated in; 1) SFM, 2) SFM + CLI-095, 3) Fetuin-A (FA), 4) FA + CLI-095, 5) CM and 6) CM + CLI-095. The final CLI-095 concentration used in the studies (10 µg/mL; ~28 µM) effectively inhibited fetuin-A uptake in these cells (data not shown). After 5 days of growth at 37°C in a humidified CO2 incubator, Alamar Blue (20 µl) was added to each well and incubated further at 37°C for 2 h. The plates were then read using a BioTek Neo2 multiplate reader (Agilent) with excitation wavelength at 560 nm and emission wavelength at 590 nm, giving as an accurate estimation of cell numbers in each well expressed as arbitrary units of fluorescence. The readings expressed as arbitrary units of fluorescence of each well were averaged + SD.

### Invasion of MDA-MB-468 is modulated by the Fetuin-A-TLR4 signaling network

Even though advances in cell and molecular biology approaches has shed light on the mechanisms of invasion and motility, there are still a number of gaps in our knowledge of the process. In most motility and invasion assays based on Boyden trans-well set up, complete medium (CM) containing 10% v/v fetal bovine serum is usually included in the bottom wells. It is then assumed that the growth factors in the fetal bovine serum such as EGF would be the chemo-attractants. We have proposed that uptake of fetuin-A (inhibited by CLI-095, a specific inhibitor of TLR4 signaling) along its concentration gradient from the bottom wells initiates the process of invasion [11]. We therefore repeated the invasion assays using parental MDA-MB-468 to assess the ability of TLR4 inhibitor to influence invasion of the cells through a bed of Matrigel. Cells (125,000 cells/well) in SFM without (control wells) or containing CLI-095 dissolved in DMSO at a final concentration of (10 µg/mL; 28 µM), were added to the upper wells (coated with Matrigel) of Boyden Chamber trans-well set-up. The control wells contained the same amount of DMSO as experimental wells. The bottom wells contained 500 µl of complete medium (CM). In order to demonstrate that it is fetuin-A in complete medium that initiated the invasion process, we repeated the experiment with complete medium depleted of fetuin-A (CM + HA) in the bottom wells. The depletion was done by incubating the medium with hydroxyapatite overnight at 4°C followed by centrifugation (5,000 x g) to remove the hydroxyapatite nanoparticles essentially as described [9].

### Statistics

Each Figure is representative of at least 3 separate experiments. Graphs and statistical analyses were generated using either Microsoft Excel or Python (numpy, scipy and matplotlib) statistical packages. P values less than 0.05 were considered significant.

## Results

### Overexpression of fetuin-A in MDA-MB-468

**Figure 1:**
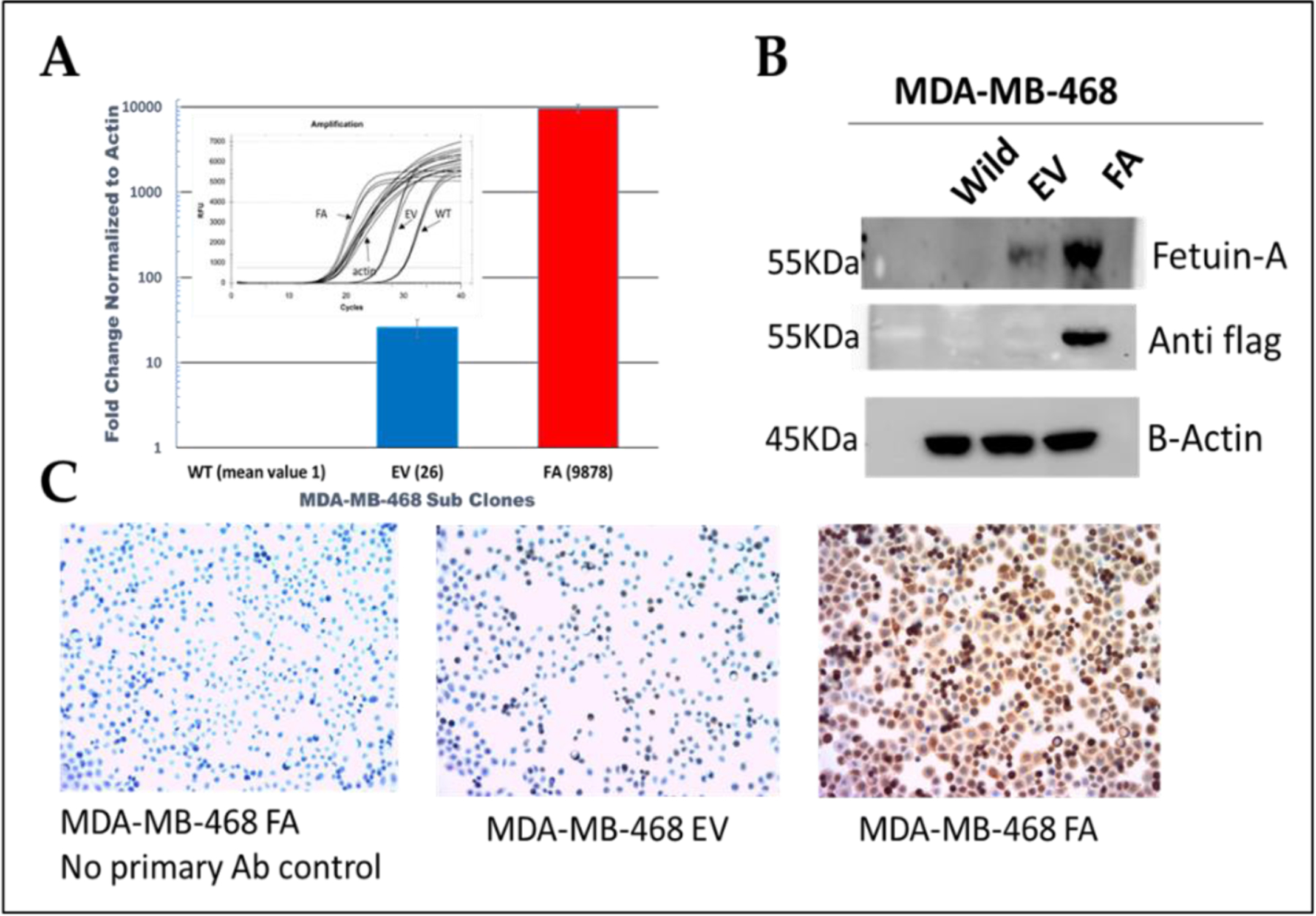
Overexpression of fetuin-A (Fet-A) in MDA-MB-468 TNBC cell line. MDA-MB-468 cells were transfected with either empty vector (EV) or flag-tagged fetuin-A expression vector (FA). Fetuin-A expression level was determined by QT-RTPCR (panel A), Western blot (panel B) and IHC (panel C) as described in Materials and Methods.

We utilized the lentiviral system to stably overexpress fetuin-A in the basal like triple negative breast cancer cell line MDA-MB-468, at the mRNA (Fig. 1A, red bar) and protein (Fig. 1B and 1C) levels. Even though Wildtype MDA-MB-468 did not show a protein band on the western blot (Fig. 1B), there was nevertheless some expression at mRNA level as shown in Fig. 1A inset. This level of expression was set at 1 and so MDA-MB-468-EV had ~10-fold higher fetuin-A expression (Fig. 1A) perhaps due to transfection manipulation. However, MDA-MB-468-FA transfected with fetuin-A expression vector had ~10,000-fold higher expression level at the mRNA level (Fig. 1A).

Because previous data from our group and others had shown that ectopically produced fetuin-A promotes adhesion and motility of other tumor cell types, we next considered assaying for invasion capacity in MDA-MB-468 cells over-expressing fetuin-A.

### Forced expression of fetuin-A in MDA-MB-468-FA increased invasion capacity of the cells relative to transfection controls and also upregulated TLR4 expression

**Figure 2:**
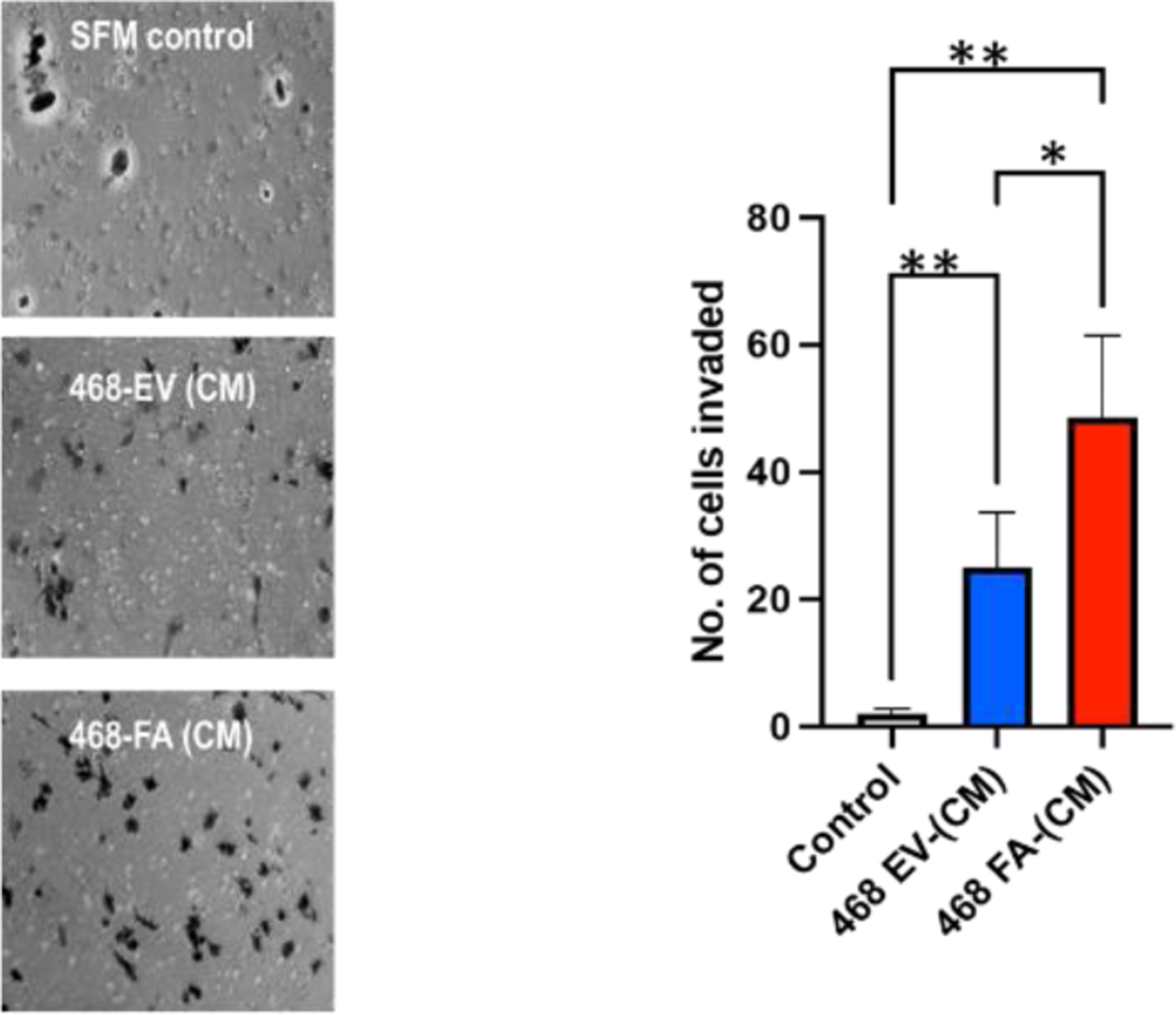
Enhanced invasion capacity in fetuin-A overexpressing MDA—MB-468 cells. The MDA-MB-468-FA invaded through a bed of Matrigel more readily compared to MDA-MB-468-EV when complete medium (CM) was in the lower compartment of the trans-well (*P<0.05); N=6). Both sub-clones did not invade when SFM was in the lower compartments (controls) (**P<0.001; N=6)

In 4 separate experiments, MDA-MB-468-FA cells were significantly more invasive as compared to their transfection controls, MDA-MB-468-EV (Fig. 2). We previously demonstrated that tumor cell lines that express high levels of ectopic fetuin-A, are more invasive, a phenotype that was attenuated when fetuin-A was knocked down in these cells [5]. We have also reported that uptake of fetuin-A culminating in adhesion in tumor cells was mediated by TLR4 [11]. Expression of TLR4 in tumor cells however varies from one cell type to the other and appears to be under tight regulatory control. For example, report by Mehmeti et al. [22] demonstrated very low almost negligible levels of TLR4 in MDA-MB-468. We also recorded very low levels of TLR4 in these cells.

**Figure 3:**
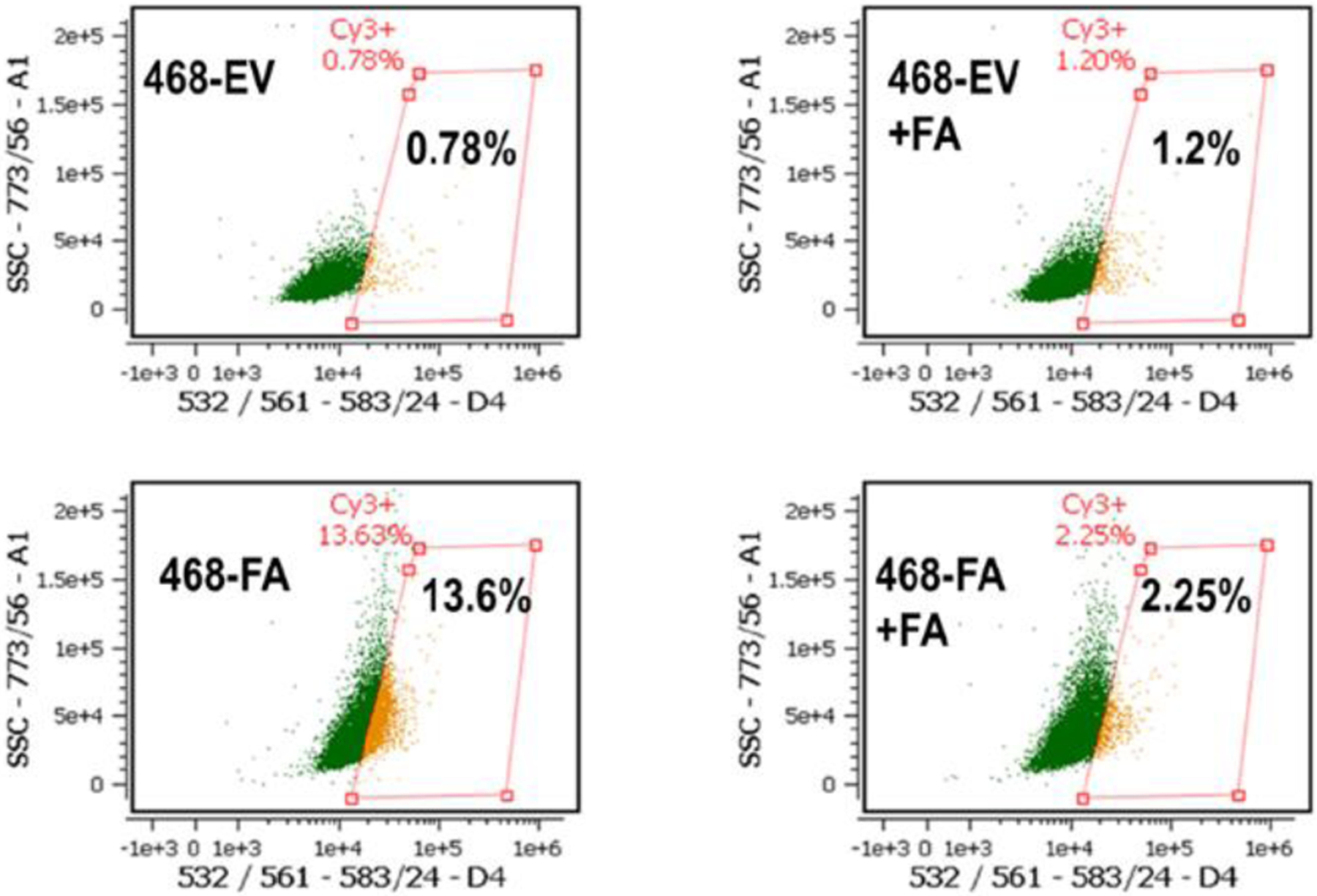
Toll like receptor 4 (TLR4) expression in MDA-MB-468 sub-clones. Surface TLR4 expression levels were determined by flow cytometry in MDA-MB-468-EV (upper panels) and MDA-MB-468-FA (lower panels) in the absence and presence of added fetuin-A (FA)

We questioned whether the expression levels of TLR4 in MDA-MB-468 could be influenced by either added fetuin-A or forced expression of fetuin-A in these cells. As expected, our data show that MDA-MB-468-EV in serum free medium, expressed very low levels of TLR4 (0.78%) as measured by flow cytometry (Fig. 3, upper panels). However, in the presence of fetuin-A (Fet-A) (2 mg/ml) in serum free medium, there was a slight increase in the surface levels of TLR4 (1.2%). The overexpression of fetuin-A mRNA in MDA-MB-468-FA in serum free medium on the other hand, dramatically increased TLR4 expression by almost 20-fold (13.4%). This high level was however attenuated in the presence of fetuin-A (2 mg/ml) to 2.25% (Fig. 3, lower panels).

### Growth of MDA-MB-468-FA in 2-dimensional (2-D) as well as 3-dimensional (3-D) is influenced by fetuin-A

**Figure 4:**
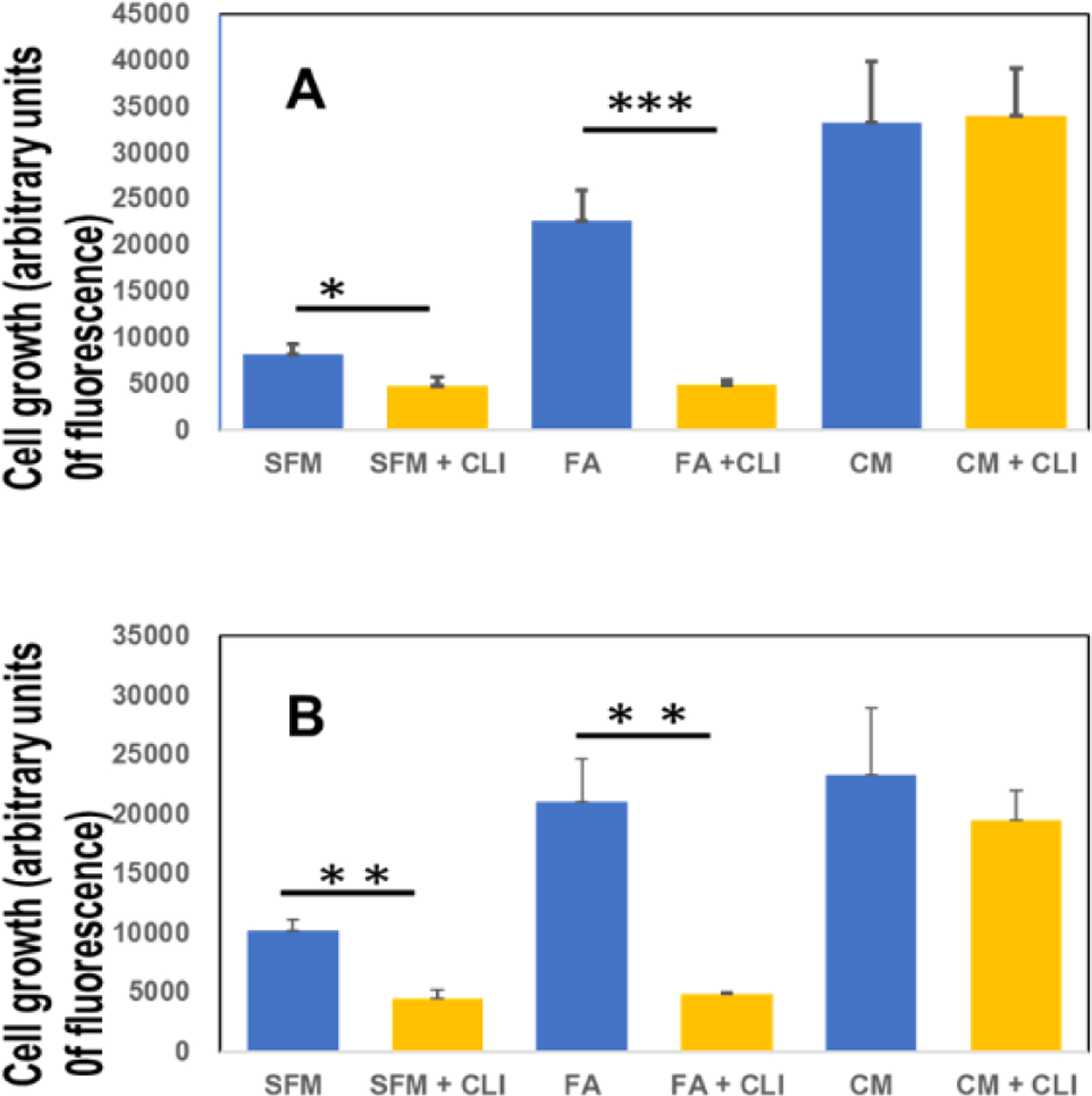
Fetuin-A mediates 2-D and 3-D growth of MDA-MB-468 via TLR4 signaling. The cells were seeded (2 × 10^4^ cells/well) in attachment (2-D) (**panel A**) or ultra-low attachment (3-D) (**panel B**) 96-well plates in SFM; SFM + CLI-095 (CLI); Fetuin-A (FA); FA + CLI; complete medium (CM); and in CM + CLI. After 6 days of growth, Alamar blue was added to each well, incubated for 2 h and fluorescence measured as described. *P< 0.05; **P< 0.001; ***P<0.0001; N= 4

In order to grow, the TNBC cells still need either the presence of fetuin-A (FA) in serum free medium or complete medium containing 10% fetal bovine serum (Fig. 4A&B). In order for fetuin-A to mediate cellular adhesion and growth in 2-D, its uptake (which can be inhibited by a specific TLR4 inhibitor) is a pre-requisite [11]. Inhibition of fetuin-A uptake attenuated cellular adhesion and growth only in those wells containing fetuin-A in serum free medium (Fig. 4A). Adhesion and growth of cells growing in complete medium which contains fetuin-A (~2 mg/ml) in addition to other adhesion molecules was not affected significantly by the TLR4 inhibitor, CLI-095 (28 µM) in 2-D cultures (Fig. 4A). There was moderate growth of cells in wells containing fetuin-A (2 mg/ml) in serum free medium but the growth was significantly inhibited by CLI-095 (Fig. 4A). As previously reported for prostate cancer cell line [8], the MDA-MB-468 cells grew well in 3-D culture wells containing fetuin-A (2 mg/ml) in serum free medium as well as complete medium (forming grapelike clusters of cells) but grew poorly in SFM (Fig. 4B). Interestingly, the 3-D growth of cells in wells containing fetuin-A in SFM was significantly attenuated by CLI-095 (28 µM) but not the 3-D growth of the cells in complete medium (Fig. 4B).

### Invasion of MDA-MB-468 cells is attenuated by the specific TLR4 inhibitor, CLI-095 cells and in the presence of complete medium depleted of fetuin-A

**Figure 5:**
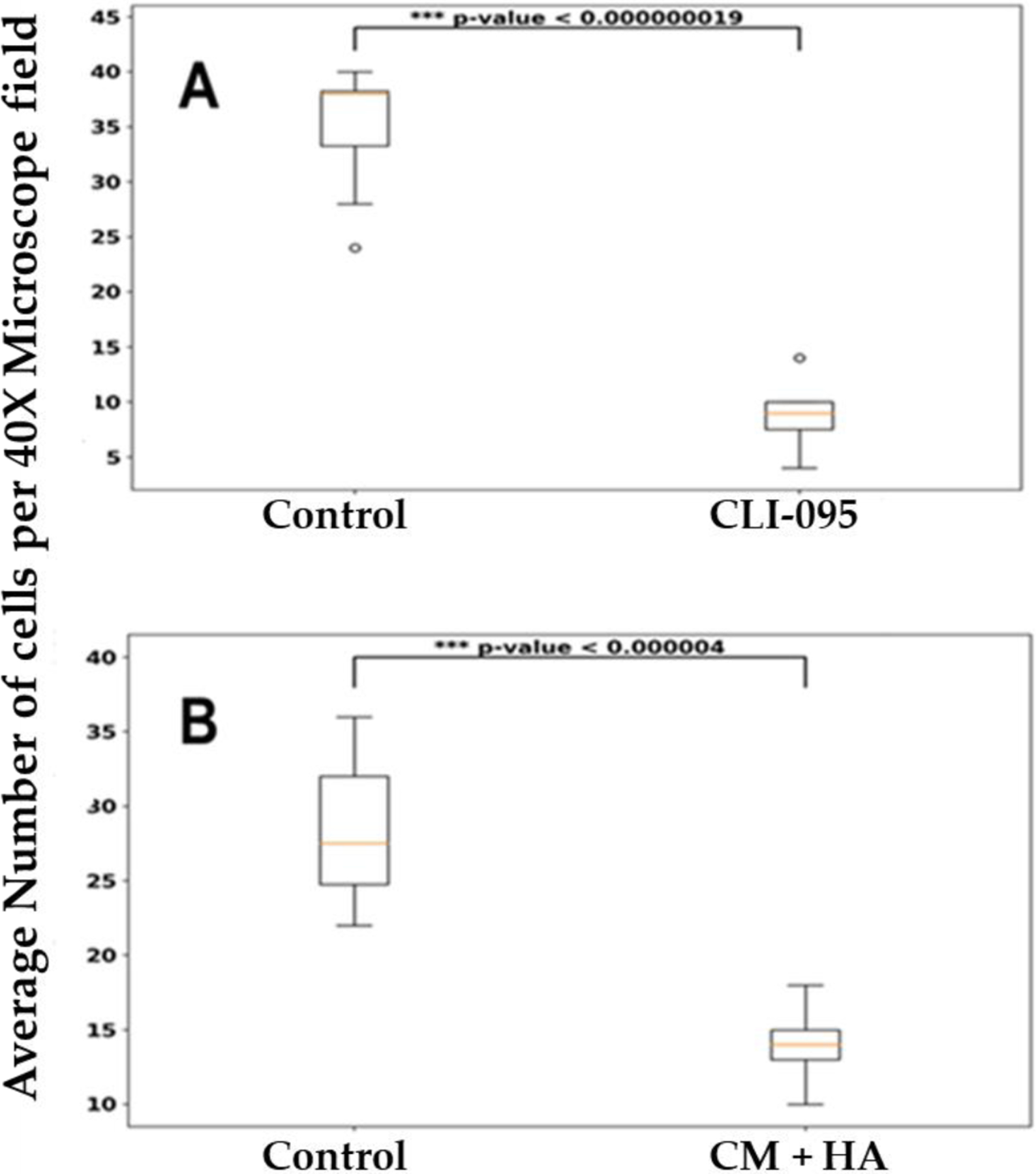
Attenuation of fetuin-A mediated invasion by inhibition of TLR4 signaling network. In **A**, control chambers had only cells in SFM, while experimental chambers had CLI-095 (28 µM) in SFM (upper chambers) of trans-well assay. The bottom chambers had complete medium (CM). In **B**, controls had CM only in the bottom chambers while experimental chambers had CM depleted of fetuin-A (CM + HA). Number of invading cells was determined as described in Materials and Methods.

Invasion of the TNBC cell line was attenuated in the presence of a specific inhibitor of TLR4, CLI-095 also known as resatorvid at a concentration of 28 µM (Fig. 5A). We previously showed this concentration of resatorvid was effective in inhibiting motility and invasion in other tumor cells [11]. We previously proposed that the major chemoattractant in complete medium is fetuin-A. This is because we could replace complete medium in the bottom wells of trans-well invasion and motility assays with fetuin-A at approximately 2 mg/ml and still maintain chemoattraction [23]. We therefore depleted L15 medium containing 10% fetal bovine serum of fetuin-A and repeated trans-well assays. We demonstrated in three separate experiments that invasion was significantly attenuated in the presence of complete medium depleted of fetuin-A (CM + HA) relative to complete medium (CM) control (Fig 5B).

## Discussion

The role that fetuin-A synthesized by cancer cells plays in tumor cell growth has been rather difficult to explain from a mechanistic viewpoint, given that the concentration of fetuin-A in the blood is relatively high and likely to supply all the needs of the growing tumor mass. Indeed, in the present studies, the ectopically produced fetuin-A was not sufficient to drive the growth of the cells in serum free medium as had been shown for other tumor cells such as glioblastoma [5]. Interestingly, glioblastoma cells also express high levels of ectopic fetuin-A and are more motile and invasive, phenotypes that were attenuated when fetuin-A in these cells was knocked down [5]. The report by Mintz et al suggested that the ectopically produced fetuin-A drove the progression of prostate cancer culminating in the metastatic spread to the bones [6]. We hereby demonstrate that overexpression of fetuin-A by the basal like triple negative breast cancer cell line, MDA-MB-468, enhances its capacity for invasion. The basal like triple negative breast cancer cell line MDA-MB-468 is an excellent prototype of the cancer it represents in that it lacks estrogen, progesterone and epidermal growth factor 2 receptors [18, 22]. Triple negative breast cancer, a type of basal breast cancer that disproportionately affect women of color has poor prognosis with fewer treatment options [24]. A recent survey of TCGA survival data (Kaplan Meir Plotter) showed that basal type breast cancers including triple negative that express high levels of fetuin-A, have worse overall survival compared to those that express low levels of the protein [21], suggesting that fetuin-A upregulates metastatic processes in these cells. Based on these studies, we overexpressed fetuin-A in MDA-MB-468 to determine if this was sufficient to confer rapid growth, adherence and spreading in these cells, cellular phenotypes that we had previously attributed to fetuin-A [5]. Overexpression of fetuin-A in MDA-MB-468 did not confer in vitro growth advantage (2-D or 3-D) to these cells (data not shown), suggesting that overexpression of fetuin-A in these cells would likewise not confer growth advantage in vivo. Nevertheless, the increased invasive capacity as well as TLR4 expression in the TNBC cells resulting from overexpression of ectopic fetuin-A, suggested that other signaling pathways that promote invasion and metastasis are also upregulated.

The basal like triple negative breast cancer cell line, MDA-MB-468 typically express very low levels of TLR4 [22]. We believe this is the first report showing that fetuin-A overexpression can upregulate TLR4 expression in these tumor cells. Even more interesting was the fact that in the transfection controls where the expression of TLR4 was almost negligible, the presence of fetuin-A at approximately 2 mg/ml was sufficient to increase surface expression of TLR4 underscoring the role of fetuin-A in the tight regulation of surface expression of TLR4 on tumor cells. A number of studies have reported the role of TLR4 in tumor motility, invasion and metastasis (13). However, the ligand that initiates TLR4 signaling in tumor cells in particular has been a subject of intense debate. Whether it is the natural ligand for TLR4 lipopolysaccharide (LPS) or some other cellular protein such as high-mobility group box-1 protein (HMGB1) [13]. Fetuin-A was originally proposed to be a ligand for TLR4 by Pal et al [25]. The publicly available protein/protein binding data (STRING) shows that other than serum albumin, TLR4 has the strongest binding interaction with fetuin-A. The present studies suggest that cell surface TLR4 is the receptor through which fetuin-A interacts with MDA-MB-468 cells. It is not unusual for a ligand to modulate the expression of its receptor and vice-versa [26, 27].

Fetuin-A supplied to the tumor cells either from the medium or blood (in vivo) modulates cellular adhesion, motility, and growth in a very elaborate mechanism. To begin with, fetuin-A has to enter the cell as a prelude to cell adhesion and cell spreading. We have reported the ability of fetuin-A to prepare a unique population of extracellular vesicles/exosomes that then promote the cellular adhesion and spreading. We have demonstrated that only those exosomes that are isolated and secreted in the presence of fetuin-A have the ability to promote adhesion and cell spreading [9]. Those prepared in the absence of fetuin-A, lack this ability [9]. In the present studies we show that fetuin-A alone is sufficient to drive the adhesion and spreading and growth of MDA-MB-468 cells both in 2-D as well as 3-D, processes that were abrogated by the specific TLR4 inhibitor. These we believe are the cells that also have the propensity for invasion.

The present data suggest that even though fetuin-A is capable of signaling growth and adhesion in MDA-MB-468 cells, other adhesion and growth signals are at play in the presence of complete medium, so the cells will use these default pathways and bypass the fetuin-A/TLR4 signaling network when it is specifically targeted by CLI-095. The fact that CLI-095 only reduced the 2-D and 3-D growth in wells containing fetuin-A but not complete medium shows that the action of CLI-095 was specific for the fetuin-A mediated adhesion and growth. Lastly the data suggest that in addition to other known chemo-attractants such as EGF, fetuin-A is the dominant chemo-attractant in complete medium (CM) that is usually added to the lower chambers in trans-well invasion and motility assays [23]. The depletion of fetuin-A in complete medium significantly attenuated the ability of CM to attract the cells as they invaded through a bed of Matrigel. Likewise, in blood vessels next to a growing tumor mass and where fetuin-A is maintained at a concentration of approximately 0.3 mg/ml, it is likely to play a role in the initial attraction of invading tumor cells during the process of intravasation.

In summary we have demonstrated that elevated expression of fetuin-A in a triple negative breast cancer cell line promotes invasion capacity of the cells and upregulates TLR4 expression in these cells. These we believe are the novel aspects of this study because, the findings could explain at least in part why TNBC which express high levels of ectopic fetuin-A have poorer prognosis. The data also suggest that fetuin-A in the medium can modulate the surface expression of TLR4 on tumor cells (either upregulation or downregulation) to promote adhesion, invasion and growth. More importantly our data show that fetuin-A is the putative ligand that signals through TLR4 to promote the growth and invasive capacity of MDA-MB-468.

## Data Availability

Data is contained within the article.
